# Supplementary material for: The application of two drainage angles in neurocritical care patients with complicated pneumonia: a randomized controlled trial
Source: PeerJ. 2024 Feb 29;12:e16997. doi: 10.7717/peerj.16997 (PMC10909356; doi:10.7717/peerj.16997)
Supplement: Supplemental Information 3 [file peerj-12-16997-s003.docx]

Trial Protocol

**Aims of the study:**

To investigate the impact of postural drainage angle after vibratory sputum removal and to evaluate the outcome of the patients, and to provide the optimal postural drainage angle for patients with neurological critical illness combined with pneumonia with minimal risk.

**Study design:**

This study aimed to implement a randomized controlled clinical trial protocol utilizing a 1:1 matched-paired design, which involved categorizing participants into experimental and control groups.

**Sample size and research plan:**

To estimate the required sample size, the PASS (Power Analysis and Sample Size) 15.0 software was employed. Based on the results of the pre-test, a Type II error (β) of 0.1 was set, corresponding to a test efficacy (1-β) of 0.9, and a Type I error (α, bilateral) of 0.05 was established. After inputting these values into the software, a sample size of 52 cases was calculated. Considering an upper limit of 20% for natural and unforeseen events impacting participant retention (such as changes in condition or discharge/transfer), a sample size of 72 cases was deemed appropriate for inclusion in this study.

**Study plan:**

The study plan is outlined as follows:

*Phase I* (2021.1-2021.5): Project preparation, including the design, discussion, and printing of the case record form, researcher's handbook, and related informed consent form. Additionally, training for the experimental staff on relevant theories and skills will be conducted. The inclusion of research subjects will commence, with 6 cases planned to be enrolled during this phase.

*Phase II* (2021.5-2021.7): Continuation of subject enrollment, with an additional 10 cases included. At this stage, the mid-term data analysis and evaluation scale will be completed, along with the mid-term summary.

*Phase III* (2021.7-2021.12): Continued enrollment of approximately 56 patients, completion of follow-up and data analysis for all enrolled patients, and final conclusion. This phase will involve a summary meeting and the writing of a research paper and report.

**Study period**

2021.1-2021.12

**Inclusion and exclusion criteria**

The inclusion criteria were as follows: 1) neurocritical care patients with artificial airways and pneumonia, aged 18–65 years, both sexes; 2) fulfilling the diagnostic criteria for pneumonia (Infection Group of the Chinese Medical Association & Respiratory Diseases Branch, 2018), including infiltrates on chest radiograph or computed tomography and clinical and laboratory findings, with a CPIS (Xu, Liu & Yu, 2015) of ≥5 points (8); 3) hemodynamically stable patients with stable intracranial pressure-related indicators (owing to differences in the patients' baseline conditions, stability was evaluated comprehensively from the following three aspects: the bone window, unchanged pupil size, and stable intracranial pressure (ICP) monitoring indicators), where a change in body position and bed-head position did not affect patient stability and stable vital signs, and with a difference of < 5% in blood pressure between both arms; and 4) after a review by the hospital ethics committee, patients or their legal representatives agreed to participate in the trial and signed informed consent forms. The exclusion criteria included the following: 1) patients with unstable conditions and a high risk of intracranial hypertension and brain herniation; 2) patients with severe liver or kidney dysfunction or severe underlying lung disease, such as chronic obstructive pulmonary disease and lung cancer; 3) patients who could not be placed in a head-tilt or head-flat position owing to limitations of their disease condition; and 4) patients who were at high risk for aspiration or had undergone esophageal, gastric, or lung resection within the past 6 months.

**Operating procedure**

The experimental and control groups underwent mechanically-assisted mucus clearance combined with percussion techniques using the following treatment process: 1) identification of lung segments that required focused interventions based on imaging examinations and auscultation. When the patient was in the lateral position, an attempt to position the target lung segments in the upper position was made. 2) Patient preparation: patients receiving tube feeding were made to pause feeding 30 min in advance. For positioning, with the patient placed in the right lateral decubitus position, the procedure was performed by two operators. The operators stood on both sides of the patient, one near the head and chest and the other near the waist and buttocks. The operator on the left side hugged the patient's shoulders and waist with both hands, whereas the other one on the right side hugged the patient’s waist and behind the knees with both hands. Subsequently, with a numbered countdown from 1 to 3, they shifted the patient to the left side of the bed, bent the patient’s knees, and turned them onto their right side. A soft pillow was placed in front of the chest, and another was placed between the legs for support. The patient's body was positioned laterally at an angle of approximately 100°. 3) An appropriate percussion head was chosen based on the patient's age and body surface area. 4) The treatment vibration frequency was adjusted to 25–30 Hz, and the vibration duration was set to 10 min. 5) Holding the handle of the percussion device with one hand, the percussion head was pressed firmly with the other hand to ensure that the percussion head was in close contact with the skin of the affected lung area on the chest wall (avoiding the spine, scapula, and kidney areas) before beginning vibrations. 6) Manual high-frequency percussion was performed on the back for 5 min (percussion frequency: 150–200 times/min (Xu, Chen & Wang, 2013). 7) Suctioning: high-flow oxygen was administered for 30 s before suctioning (2 min for patients receiving mechanical ventilation with 100% oxygen concentration) using a closed suction system matched to the patient's endotracheal/tracheostomy tube size. The suction pressure was set to 20–26.7 kpa (Shen & Xia, 2004). Postural drainage was performed after mucus suction. The control group individuals were placed supine with the head of the bed at 0° for drainage (the bed head angle was changed from 30° to 0° at a rate of 5°/2 min to allow the patient to adapt to the position). The experimental group patients were positioned with the head of the bed tilted 30° for drainage. Both groups were maintained in their respective positions for 30 min. Notably, if the patient had a ventricular drainage tube, it was opened during postural drainage, and the preset height of the drainage tube adjusted accordingly. In case the lateral position was related to the location of the patient's lung lesion and could not be uniformly auto-positioned, the blood pressure of the opposite upper limb was measured in the lateral position, and the opposite upper limb was placed on a soft pillow and elevated near the heart level.

**Research instruments**

A Spiegelberg ICP monitor was used. 2) A Mindray T8 electrocardiogram monitor was used to monitor the vital signs of heart rate (HR), systolic blood pressure (SBP), diastolic blood pressure (DBP), mean arterial pressure (MAP), and oxygen saturation (SpO2). 3) Electric beds with protractors for precise head angle measurements. 4) A medical flutter mucous-clearing device (Model YS8001) for mechanically-assisted mucus clearance. 5) A soft pillow to fix the patient's position when they turned to their side to increase their comfort.

**Evaluation indicators**

SBP, DBP, MAP, HR, and SPO2 were recorded after 30 min of postural drainage in the two positions. The mucus in the main airway was removed by suction after postural drainage in both positions. Blood gas analysis was performed 30 min later . The pH, arterial oxygen pressure (PaO2), and oxygenation indices were recorded. Laboratory indicators (C-reactive protein and white blood cell count were recorded before and after the intervention), and the CPIS was calculated .

**Statistical methods**

Data were analyzed using IBM SPSS Statistics for Windows, version 26.0. For the general information in the study, continuous and categorical variables were analyzed using independent-sample t-tests and chi-square tests, respectively. Within-group comparisons across the two groups before and after the intervention were performed using paired-sample t-tests or paired non-parametric tests. Between-group comparisons across the two groups were performed using independent sample t-tests or non-parametric tests. Furthermore, variations in physiological indicators in repeated measurements were analyzed using two-way repeated measures analysis of variance (ANOVA).

[**Technology roadmap**](javascript:;)

**The inclusion criteria were as follows:**

1) neurocritical care patients with artificial airways and pneumonia, aged 18–65 years, both sexes; 2) fulfilling the diagnostic criteria for pneumonia, including infiltrates on chest radiograph or computed tomography and clinical and laboratory findings, with a CPIS of ≥5 points; 3) hemodynamically stable patients with stable intracranial pressure-related indicators 4) patients or their legal representatives agreed to participate in the trial and signed informed consent forms.

**The exclusion criteria included the following:**

1) patients with unstable conditions and a high risk of intracranial hypertension and brain herniation; 2) patients with severe liver or kidney dysfunction or severe underlying lung disease; 3) patients who could not be placed in a head-tilt or head-flat position owing to limitations of their disease condition; 4) patients who were at high risk for aspiration or had undergone esophageal, gastric, or lung resection within the past 6 months.

Neurological critical illness concomitant with pneumonia

Inclusion and exclusion criteria

Patient/legal representative signs informed consent form

Cases included

Randomized controlled clinical trial protocol utilizing a 1:1 matched-paired design

random allocation

[experimental group](javascript:;)

[control group](javascript:;)

positioned with the head of the bed tilted 30° for drainage

placed supine with the head of the bed at 0° for drainage (the bed head angle was changed from 30° to 0° at a rate of 5°/2 min to allow the patient to adapt to the position).

Until the end of treatment

X-rays and laboratory tests after 3 and 7 days

No improvement in indicators after 7 days

Improved indicators after 7 days
